# Critical Thinking Dispositions of Nursing Students in Asian and Non-Asian Countries: A Literature Review

**DOI:** 10.5539/gjhs.v5n6p172

**Published:** 2013-09-26

**Authors:** Mahvash Salsali, Mansooreh Tajvidi, Shahrzad Ghiyasvandian

**Affiliations:** 1 School of Nursing and Midwifery, Tehran University of Medical Sciences, Tehran, Iran; 2 School of Nursing and Midwifery, Tehran University of Medical Sciences, International Campus, Tehran, Iran

**Keywords:** critical thinking, disposition, nursing student, literature review

## Abstract

Critical thinking disposition represents an inclination of a person to use possessed skills in relation to critical thinking. The trend of critical thinking has been described as inner motivation to solve problems and make decisions by thinking. In nursing as a practical profession, the concept of critical thinking dispositions is important component in helping to manage complex health situations and to deal with patient issues effectively. Willingness to think critically is a prerequisite for safe and subtly performance. The results of studies show critical thinking dispositions of nursing students in Asian countries are different from non-Asian countries. Aim of this literature review was to compare critical thinking dispositions of nursing students in Asian and non-Asian countries. Literature review was done in English and Persian databases. The results showed of the 795 articles published in English and Persian language that studied critical thinking, 73 ones studied critical thinking skills and dispositions in nursing education, and relationship between teaching methods and critical thinking skills and dispositions in nursing education of different countries. Fifteen of seventy three articles assessed critical thinking dispositions in nursing students. Limited studies showed that the Asian nursing students had mostly undermining score of the critical thinking dispositions, while non-Asian countries tend to positive scores. The reasons for these differences could be due to issues such as environmental, educational methods and cultural differences. However, future studies should measure critical thinking disposition by discipline-based tools.

## 1. Introduction

Literature on critical thinking shows that there is little agreement about the concept of critical thinking. Dewey (1910), from a philosophical view, proposed that critical thinking involves probing, discriminating and testing ideas as well as exploring various options on an issue. Paul (1992) described critical thinking as the ‘art of thinking about your thinking while you are thinking in order to make your thinking better; more clear, more accurate and more defensible’ (p. 11). In another study by Paul (1993), three essential elements—thought, intellectual standards, and affective traits—were identified as central to the critical thinking process. Ennis (1987) and Halpern (1998) defined the term critical thinking as represents a set of cognitive skills and dispositions which are conducive to decision making and problem solving in different situations. Watson and Glaser (1980) defined critical thinking as a composite of attitudes, knowledge, and skills. According to Facione, Facione, and Sanchez (1994), a critical thinking disposition represents an inclination of a person to use possessed skills in relation to critical thinking. The trend of critical thinking has been described as inner motivation to solve problems and make decisions by thinking (Huan & Vickie, 2008). According to American Society of Philosophy (1990) that has proposed a precise consensus on the definition of critical thinking which comprises emotional and cognitive components, ‘critical thinking is purposeful, self-regulatory judgment which results in interpretation, analysis, evaluation and inference as well as explanation of the evidential conceptual, methodological, criteriological or contextual considerations upon which that judgment was based. The ideal critical thinker is habitually inquisitive, well informed, trustful of reason, open-minded, flexible, fair minded in evaluation, honest in facing personal biases, prudent in making judgements, willing to reconsider, clear about issues, orderly in complex matters, diligent in seeking relevant information, reasonable in the selection of criteria, focused in inquiry, and persistent in seeking results which are as precise as the subject and the circumstances of inquiry permit’ (p. 3). The good critical thinker must foster critical thinking dispositions, as well as the development of critical thinking skills (Paul, 1992; Facione et al., 1994; Norris, 1995).

In nursing as a practical profession, where rapid changes occurred and decision-making is imperative, the concept of critical thinking is important portion in helping to manage complex health situations and to counter patient problems effectively (Distler, 2007; Beckie, Lowry, & Barnett, 2001; Yeh & Chen, 2003; Hwang and et al., 2010). Intending to think critically is a prerequisite for safe and subtle performance (Alfaro-LeFevre, 1999). Overall, emotional tendency toward critical thinking is a pivotal component for cognitive skills. Emphasis on cognitive skills alone without emotional attitude probably conduces the tendency to educate nurses to be closed mind, inflexible and prejudicial (American Philosophical Association, 1990). Despite the importance of critical thinking dispositions in nursing students and the necessity of its existence to reach the critical thinking performance, few studies have been conducted in this area. While critical thinking in nursing has been studied for almost 25 years, literature on critical thinking dispositions has only been within the past 10 years (Stewart, 2005). There is little research directly examining differences in critical thinking between Asian students and their non-Asian counterparts (Lun, Fischer, & Ward, 2010). Ten Dam and Volman (2004) observed, research which addresses the role of culture in relation to critical thinking is currently limited. Since the results of studies show critical thinking dispositions of students in Asian countries are different from other countries, this hypothesis would suggest that critical thinking dispositions is associated with cultural and contextual issues. (Profetto-McGrath, 2003; Ip et al., 2000; Tiwari, Avery, & Lai, 2003). Therefore the purpose of this review is the comparison of critical thinking dispositions between nursing students in Asian and non-Asian countries.

In this review, the following questions are answered: 1. What is the level of the critical thinking dispositions of nursing students in Asian countries in the literature? 2. What is the level of the critical thinking dispositions of nursing students in non-Asian countries in the literature? 3. Are there differences between critical thinking dispositions in Asian and non-Asian countries?

## 2. Method

All major electronic sources of relevant information were systematically searched to identify peer-reviewed English and Iranian language abstracts or papers published between 1990 and 2012. To identify all relevant studies for the review, the search strategy comprised searches of the following: CINAHL database, Proquest, Pub-Med, Medline, Science Direct, OVID and Iranian Journal Full-text Databases including SID, Iran medex and Magiran; Internet source (www.google.com) using the key words: ‘critical thinking’, ‘nursing’, ‘students’ ‘nursing students’ and ‘critical thinking dispositions’. Initially 717 English articles and 78 Persian articles were found. However, several were discarded as they did not specifically address critical thinking dispositions in nursing students. The inclusion criteria were (1) articles that were related to critical thinking dispositions in nursing students (2) articles that used California Critical Thinking Disposition Inventory (CCTDI) to measure critical thinking dispositions in nursing students. A variety of instruments have been used to study critical thinking in nursing education. Facione and Facione (1992) developed the California critical thinking disposition inventory (CCTDI) that was used to measure critical thinking dispositions. California Critical Thinking Disposition Inventory contains 75 items with a Likert scale ranging from completely agree to completely disagree scoring. Maximum and minimum values of 70 and 420 are obtained from this test. Strong and stable disposition (over 350), positive (between 280 and 350), undermining (between 210 and 280) and negative (below 210) are classified. The questions included seven subscales: truth-seeking (12 items), open-mindedness (12 items), analyticity (11 items), systematicity (10 items), critical thinking and self-confidence (10 items), inquisitiveness (10 items) and maturity (10 items). Total points from the seven sub-scales determine an individual’s critical thinking disposition. Therefore authors studied abstracts of 717 English articles and 78 Persian articles. English and Persian language studies were separately classified and reviewed. In the final stage, critical thinking dispositions in nursing students was compared in Asian and non-Asian countries.

## 3. Results

Of the 795 articles published in English and Persian language, 73 articles studied critical thinking skills and dispositions in nursing education, and relationship between teaching methods and critical thinking skills and dispositions in countries such as Iran, Korea, Turkey, China, Japan, Hong Kong, Jordan, Norway, United State, Canada, and Australia. Fifteen of seventy three articles assessed critical thinking dispositions in nursing students (Table 1). Studies showed differences in total scores of California Critical Thinking Disposition Inventory. Review of these articles showed only four of Iranian articles studied critical thinking dispositions in nursing students. The two of them have been conducted by the same authors in the same place. The results of these studies showed a low level of critical thinking disposition in nursing students that indicate undermining score (210-280) and no one had a positive or stable critical thinking disposition (>280) (Barkhordary, Jalalmanesh, & Mahmoodi, 2009, 2011). Also according to the results of Ranjbar and Esmaili (2006), students’ critical thinking disposition was negative (<210). Gharib (2006) examined critical thinking dispositions of nursing students in Iran University of Medical Sciences, results showed that the mean score of critical thinking disposition was 278.6 that indicated undermining attitudes toward critical thinking. Review of studies in other countries shows that the dimension of the critical thinking disposition has been given more attention than Iran. Furthermore, some studies compared critical thinking disposition of nursing students in Asian and non-Asian countries. Candan, Gonca and Aklime (2008) concluded Turkish students’ critical thinking disposition scores were 255.8 ± 23.7 (undermining attitude). Students’ critical thinking disposition scores in Hong Kong has been reported 264.70±24.01 indicating the undermining attitude of the students towards critical thinking (Ip et al., 2000). Comparison of Australian and Hong Kong students’ critical thinking disposition scores showed that Hong Kong students’ score was 268.36 (undermining attitude) and Australian students’ score was 287.73 (positive attitude) (Tiwari et al., 2003).

**Table 1:** Critical thinking disposition of nursing students in Asian and non-Asian countries

Study	Sample	CCTDI Scores
Ip et al. (2000)	Hong Kong students	264.70±24.01
Tiwari et al. (2003)	Hong Kong students Australian students	268.36 287.73
Zhang Huan & Vicki (2008)	Chinese students	272.82
Suliman and halebi (2007)	Jordanian students	284.93
Kyungrim et al. (2006)	Korean students	263
Min-Ling & CIA Sing (2003)	Chinese students American students	283 303.24
Candan et al. (2008)	Turkish students	255.8 ± 23.7
Kavashima and Petrini (2004)	Japanese students	273.38
Barkhordary et al. (2009, 2011)	Iranian students	210-280
Ranjbar and Esmaili (2006)	Iranian students	<210
Gharib (2006)	Iranian students	278.6
Giancarlo and Facione (2001)	American students	301
Wangenstein et al. (2010)	Norwegian students	300.3
McGrath (2003)	Canadian students	280- 350

**CCTDI Scores**: stable (over 350), positive (between 280 and 350), undermining (between 210 and 280) and negative (below 210)

Suliman and Halebi (2007) studied critical thinking disposition in nursing students in Jordan. The mean total score of critical thinking disposition in their study was 284.93 (positive attitude). Kyungrim, Duk, Sujin and Myoung (2006) studied critical thinking skills and dispositions in Korean nursing students in associate, bachelor’s and master’s programs that results showed the average score was 263 (undermining attitude). Huan and Vicki (2008) studied the critical thinking disposition and learning styles of baccalaureate nursing students in China, results showed that score of critical thinking disposition in Chinese students was 272.82 (undermining attitude). McGrath (2003) studied disposition of critical thinking in the Canadian nursing students. Results showed score of Canadian students’ critical thinking disposition was between 280 and 350 (positive attitude). Findings of Giancarlo and Facione’ (2001) study showed critical thinking disposition in American students was 301 (positive attitude). Wangensteen et al. (2010) studied critical thinking disposition in newly graduated nursing students in Norway; results showed that almost 80 percent of students had positive attitude toward critical thinking (score was 300.3).

Furthermore some studies showed differences in some subscales of California Critical Thinking Disposition Inventory. For example Min-Ling Yeh Chen and CIA Sing Chen (2003) compared disposition of critical thinking between Chinese and American graduate nursing students; results showed Chinese students had trends to an open-mindness, analysicity, self confidence and inquisitiveness. American students had trends to open-minded, analysicity, self confidence, inquisitiveness, systematicity and maturity. There were significant differences between the two groups in subscales of truth seeking, open-mindness and maturity. American students had a higher score of critical thinking disposition subscales than Chinese students. Authors tend to express that one reason for this difference in some subscales of critical thinking disposition comes from the cultural differences between the two groups.

According to Huan and Vicki (2008) in China, results showed between scores of critical thinking disposition subscales that were reported, truth seeking subscale score was lowest. Similar results have been noticed in other studies (Ip et al., 2000; Li & Guo, 2004). Results of study in Norwegian nursing students showed the highest score in subscale of inquisitiveness and lowest score in truth seeking (Wangensteen et al., 2010). The results of these studies show that it is essential to investigate the reasons for different nursing students’ critical thinking disposition in different countries.

## 4. Discussion

In this literature review, nursing students’ critical thinking dispositions in Asian and non-Asian countries were reviewed and compared. Limited studies showed that the critical thinking dispositions of Asian nursing students are mostly undermining. Some Asian countries such as Iran, China, Japan and Hong Kong reflect a low critical thinking disposition scores of their students (Huan & Vickie, 2008, Ip et al., 2000; Tiwari et al., 2003); while non-Asian countries tend to score highly in critical thinking dispositions in nursing students. The reasons for the low critical thinking disposition in most of studies in the Asian countries could be due to issues such as environmental, educational methods and cultural differences (Min-Ling Yeh Chen & CIA Sing Chen, 2003; Profetto-McGrath, 2003). Brown (1998) suggested that critical thinking is specific to a certain kind of culture. This may explain the observed differences in scores. Academics often affirm that Asian students do not naturally take part in critical thinking because they do not obviously contribute in classroom discussions (Paton, 2005).

In a culture that does not value critical questioning and having inconsistent viewpoints, for example in the Chinese culture (Gabrenya & Hwang 1996), students may easily lose opportunities to practise how to express conflicting views and when to delay judgment or come to a closure. Most likely the low critical thinking disposition scores of Asian nursing students are due to the application of traditional methods in nursing education, which shows the need to revise the nursing curriculum. Tiwari et al. (2003) attributed higher scores of the critical thinking disposition in Australian nursing students than those of Chinese students to the active educational model used in the Australian school and to cultural differences. Critical thinking in nursing students develops through active educational programs. Pang et al. (2004) have argued that nursing is conceptualized differently by Eastern and Western nurses. For example Chinese nursing is grounded in a cultural understanding of health. It is feasible that differences in modes of thinking between nursing students in the East and West are responsible for the difference in scores.

Furthermore, as already mentioned, studies show that nursing students in Asian countries and non- Asian countries have differences in subscales of critical thinking disposition. Most studies have reported score of truth seeking subscale in students are low. Colucciello (1997) proposed that a lower score of truth seeking could be due to traditional teaching and learning strategies where the extensive information was presented in the lectures, students are expected to learn rather than seeking information or asking questions. Huan and Vickie (2008) express Chinese students are not encouraged to ask teachers questions because teachers fear that they cannot answer questions from students and be shamed. It is another reason for the low scores of truth seeking in Chinese students. The low score in open-mindness in some studies may be due to students that could not tolerate divergent views because they are not encouraged to ask questions of the instructor or other students. Low scores on the Order-taking and analysis can also show the weakness of organized reasoning and focus on problem solving (Huan & Vickie, 2008). In general, review of studies shows that current nursing programs will not lead to a shift in critical thinking (Stephanie et al., 2005). This has raised the need to revise these programs.

Finally, the reviewed studies have applied a general tool, regardless of cultural and linguistic differences to measure critical thinking dispositions in nursing students. It could be the other reason for inconsistency in the results of studies. Many researchers have acknowledged this issue as well (Huan & Vickie, 2008; Yeh, 2003; Shin et al., 2006); Profetto-McGrath, 2003; Ip et al., 2000; Tiwari et al., 2003). There is a need to develop a discipline-specific tool designed to measure nurses’ critical thinking (Hartley & Aukamp, 1994). California Critical Thinking Disposition Inventory may be appropriated to Western social customs and beliefs that Eastern nurses cannot fully understand it.

## 5. Conclusion

According to present study, there are the diverse and often contradictory results about critical thinking dispositions of nursing students in Asian and non-Asian countries. In some cases, it has been expressed critical thinking disposition in Asian countries is lower than in other countries. It should be noted the most studies in critical thinking disposition of nursing students were descriptive studies without analyzing causes of low critical thinking disposition. Therefore following these few articles, we could not conclude that all nursing students in Asian countries have low disposition toward critical thinking. It is suggested to compare the trend of critical thinking of nursing students in different countries according to different cultural backgrounds. However, future studies should measure critical thinking disposition by discipline-based tools.
